# Histological Outcomes of Alveolar Ridge Preservation Versus Spontaneous Healing Following Tooth Extraction: A Systematic Review and Meta-Analysis

**DOI:** 10.3390/dj13120556

**Published:** 2025-11-25

**Authors:** Ioanna Benekou, Ioannis Fragkioudakis, Georgios S. Chatzopoulos, Ioannis Vouros

**Affiliations:** 1 Department of Prosthodontics, School of Dentistry, Aristotle University of Thessaloniki, 54124 Thessaloniki, Greece; 2 Department of Preventive Dentistry Periodontology and Implant Biology, School of Dentistry, Aristotle University of Thessaloniki, 54124 Thessaloniki, Greece; ifragkio@dent.auth.gr (I.F.); gschatzo@dent.auth.gr (G.S.C.); iovouros@dent.auth.gr (I.V.)

**Keywords:** alveolar ridge preservation, socket preservation, histomorphometry, bone graft, new bone formation

## Abstract

**Objectives:** This systematic review and meta-analysis aimed to evaluate histological outcomes of alveolar ridge preservation (ARP) compared to spontaneous healing after tooth extraction, focusing on the percentage of new bone formation and residual graft material. **Materials and Methods**: Randomized controlled trials (RCTs) assessing histomorphometric outcomes in humans were included. Eligible studies compared ARP using various graft materials to unassisted socket healing. Primary outcome was new bone formation (%); secondary outcome was residual graft material (%). Random-effects meta-analyses and subgroup analyses were conducted based on graft material type, membrane application, and healing duration. Risk of bias and certainty of evidence were assessed using the Cochrane RoB 2.0 tool and GRADE, respectively. **Results**: Twenty-two RCTs (816 patients) met inclusion criteria. Overall, ARP did not result in significantly greater new bone formation compared to spontaneous healing (mean difference −5.86%, 95% CI: −13.84% to 2.11%, *p* = 0.15; I^2^ = 98%), revealing a paradoxical trend: while ARP maintains ridge volume, histologically it may yield slightly lower proportions of vital bone compared to unassisted healing. However, autologous biomaterials (e.g., PRF/CGF) and xenografts with collagen membranes showed significantly new bone formation (mean differences +16.28% and −22.47%; *p*< 0.001 and *p* = 0.003, respectively). Residual graft content was highest in relation to xenografts and allografts, particularly without membranes. Long-term studies demonstrated a statistically significant benefit for ARP following excluding high-risk trials. **Conclusions:** Histological advantage of ARP is biomaterial-dependent. Autologous platelet concentrates and xenografts with membranes yielded the most consistent bone regeneration outcomes. Clinical Relevance: Although ARP preserves socket volume, its histological superiority over natural healing is not universal. Therefore, the selection of biomaterials and determination of and appropriate healing period are critical parameters.

## 1. Introduction

Tooth extraction is an indispensable procedure in clinical dentistry, commonly performed due to advanced caries, periodontal disease, endodontic failures, or trauma. However, the biological consequence of extraction is a substantial resorption of the alveolar ridge, which can significantly compromise future prosthetic rehabilitation, particularly the placement and esthetic success of dental implants [1,2]. The loss of alveolar bone is most pronounced within the first few months after extraction, with studies demonstrating that approximately two-thirds of the total reduction in ridge volume occurs within the first 12 weeks [3,4]. The buccal plate, being thinner and composed largely of bundle bone, is especially susceptible to rapid resorption, particularly in the esthetic anterior maxilla [5,6].

To counteract these dimensional changes, various socket preservation techniques—also termed alveolar ridge preservation (ARP)—have been developed. ARP involves placement of grafting materials, often combined with barrier membranes, into the socket immediately after tooth extraction, aiming to mitigate hard and soft tissue collapse [7,8]. The biological rationale behind ARP is to provide a scaffold for bone ingrowth, maintain space, and promote clot stability, thereby modulating the natural healing cascade [1]. A wide range of biomaterials has been investigated for this purpose, including xenografts, allografts, synthetic bone substitutes (alloplasts), demineralized bone matrices, and autologous platelet concentrates, each characterized by distinct osteoconductive and osteoinductive properties [9,10].

Although ARP has been shown to limit dimensional shrinkage compared to spontaneous healing, its histological efficacy, specifically in promoting new vital bone formation versus the presence of residual biomaterials, remains debated [11,12]. Some materials, particularly slow-resorbing xenografts, have been associated with significant amounts of residual graft material even months after placement, which could influence subsequent implant osseointegration [10,13]. In contrast, autologous approaches such as platelet-rich fibrin (PRF) are hypothesized to accelerate natural bone regeneration with minimal foreign body persistence [14].

Despite numerous randomized controlled trials (RCTs) evaluating various ARP approaches, inconsistencies in study designs, grafting protocols, histological methodologies, and reporting standards have complicated direct comparisons. Moreover, existing systematic reviews have either focused primarily on clinical dimensional outcomes (e.g., ridge width, height) or included heterogeneous study designs without stratifying by histological parameters [8,15]. A focused and methodologically rigorous synthesis of histological data from RCTs is therefore urgently needed to clarify the true regenerative capacity of different socket preservation strategies.

The primary objective of this systematic review and meta-analysis is to evaluate the histological outcomes of alveolar ridge preservation (socket preservation) compared to spontaneous healing after tooth extraction in human subjects, focusing on new bone formation and residual graft material proportions. Secondary objectives include investigating whether different graft material types (xenografts, allografts, alloplasts, autologous concentrates) and the use of barrier membranes influence these histological outcomes. By systematically synthesizing and quantitatively analyzing data from randomized controlled trials, this review aims to identify the most biologically effective strategies for socket preservation, thereby informing clinical protocols and improving implant treatment predictability.

## 2. Materials and Methods

### 2.1. Protocol and Registration

This systematic review and meta-analysis were designed and conducted in accordance with the Preferred Reporting Items for Systematic Reviews and Meta-Analyses (PRISMA 2020) guidelines. The protocol was prospectively registered in the International Prospective Register of Systematic Reviews (PROSPERO) under the registration number CRD420251019531. The PRISMA 2020 Checklist is available in Supplementary Table S1. The review was developed according to the PRISMA-PICOS framework, defining eligibility based on study population, intervention, comparator, outcomes, and study design.

In accordance with PRISMA guidelines, the focused PICO question was formulated as follows:

Population (P): patients undergoing tooth extraction;
Intervention (I): alveolar ridge preservation (ARP) using any graft material and/or membrane;
Comparison (C): spontaneous (unassisted) socket healing;
Outcome (O): histomorphometric parameters, including percentage of new bone formation, residual graft material, and connective or non-mineralized tissue.

### 2.2. Eligibility Criteria

Studies were eligible if they fulfilled the following inclusion criteria: (1) randomized controlled trials (RCTs) in human subjects who underwent tooth extraction; (2) intervention involving alveolar ridge preservation (ARP) using any grafting material, including xenografts, allografts, synthetic bone substitutes (alloplasts), demineralized bone matrix, or autologous platelet concentrates; (3) comparison to spontaneous healing (i.e., healing without the use of grafts or membranes); (4) quantitative reporting of histological or histomorphometric outcomes such as percentage of new bone formation, residual graft material, or connective tissue; and (5) minimum follow-up duration of two months post-extraction.

Exclusion criteria comprised the following: (1) non-randomized studies (e.g., case series, cohort studies), (2) animal or in vitro studies, (3) studies lacking histological endpoints or without a spontaneous healing control group, and (4) studies incorporating additional interventions beyond socket preservation (e.g., guided bone regeneration, simultaneous implant placement).

### 2.3. Information Sources

A comprehensive literature search was conducted across five electronic databases: PubMed/MEDLINE, EMBASE, Scopus, Web of Science, and the Cochrane Central Register of Controlled Trials (CENTRAL). The final search was completed on April 15, 2025. Additional manual searches were performed on the reference lists of included studies and relevant systematic reviews to ensure completeness.

### 2.4. Search Strategy

Search strategies were customized for each database using a combination of Medical Subject Headings (MeSH) and free-text terms. Keywords included “tooth extraction,” “socket preservation,” “alveolar ridge preservation,” “bone graft,” “xenograft,” “allograft,” “synthetic bone substitute,” “histological analysis,” “new bone formation,” and “randomized controlled trial.” Full search strategies for each database are detailed in the Appendix A Table A1.

### 2.5. Selection Process

Two independent reviewers (I.B. and I.F.) screened all titles and abstracts for eligibility. Full texts of potentially relevant articles were then retrieved and evaluated against the inclusion and exclusion criteria. Inter-reviewer agreement was assessed using Cohen’s kappa coefficient, which demonstrated substantial agreement (κ > 0.80). Any discrepancies were resolved through discussion, and where needed, a third reviewer (I.V.) was consulted to reach consensus. This process ensured consistency and reproducibility in study selection.

### 2.6. Data Collection Process

Data extraction was conducted independently and in duplicate by two reviewers using a standardized electronic form, in accordance with Cochrane guidelines. Extracted data included study characteristics (author, year, country, design), sample size, participant demographics, grafting material type, membrane application, healing duration, histological evaluation method, and histomorphometric outcomes (new bone formation percentage, residual graft, connective tissue). For studies comparing multiple interventions against a single control group, we adhered to the Cochrane-recommended strategy of combining the experimental arms to prevent unit-of-analysis errors. In cases where relevant data were missing, unclear, or inconsistently reported, attempts were made to contact study authors via email to obtain clarification or additional information. All discrepancies in extracted data or interpretations were resolved through discussion between the two reviewers. If consensus was not reached, a third reviewer was consulted to adjudicate the decision. Extraction was conducted independently and in duplicate by two reviewers using a standardized electronic form, in accordance with Cochrane guidelines. Extracted data included study characteristics (author, year, country, design), sample size, participant demographics, grafting material type, membrane application, healing duration, histological evaluation method, and histomorphometric outcomes (percent new bone, residual graft, connective tissue). For studies comparing multiple interventions against a single control group, we adhered to the Cochrane-recommended strategy of combining the experimental arms to prevent unit-of-analysis errors. Any inconsistencies were resolved through consensus or third-party adjudication.

### 2.7. Data Items

Primary outcome: percentage of new bone formation within the socket, measured histologically. Secondary outcomes included percentage of residual graft material, percentage of connective or trabecular tissue, graft material category, membrane application, and healing time.

### 2.8. Risk of Bias Assessment

The risk of bias was independently assessed for all included RCTs using the Cochrane Risk of Bias 2.0 (RoB 2) tool. This structured tool evaluates five domains: (1) bias from the randomization process, (2) bias due to deviations from intended interventions, (3) bias due to missing outcome data, (4) bias in outcome measurement, and (5) bias in selection of the reported result. Judgments were categorized as low-risk, some concerns, or high-risk. Ten studies were judged as low-risk, six had some concerns, and five were deemed high-risk. Such studies were appraised with particular scrutiny in the RoB 2 assessment and incorporated into sensitivity analyses to evaluate their impact on the overall findings. Ratings were also integrated into the GRADE assessment framework to assess confidence in effect estimates.

### 2.9. Effect Measures

All outcomes were continuous and reported as mean differences (MDs) with corresponding 95% confidence intervals (CIs). When studies reported data as medians and ranges or interquartile intervals, these were converted to means and standard deviations using the method proposed by Hozo, Djulbegovic, and Hozo (2005) [16], which provides an established approach for approximating parametric estimates from nonparametric summary data. This approach has been widely adopted in meta-analyses involving continuous outcomes.

### 2.10. Synthesis Methods

Meta-analyses were conducted using a random-effects model (DerSimonian and Laird method) to account for expected clinical and methodological heterogeneity. In studies with multiple intervention arms compared against a single control group, we combined intervention groups to avoid unit-of-analysis errors, as recommended by the Cochrane Handbook. Heterogeneity was assessed using the I^2^ statistic, with 25%, 50%, and 75% interpreted as low, moderate, and high heterogeneity, respectively. Tau^2^ (τ^2^) was also calculated to estimate between-study variance. Influence diagnostics including DFFITS, Cook’s Distance, and leave-one-out sensitivity analyses were conducted to identify outliers and assess model robustness.

Meta-analytic computations were performed using Review Manager (RevMan Web, Cochrane Collaboration, London, United Kingdom, 2025), and JASP version 0.18.2. Forest plots were generated to present individual and pooled effect sizes, and all analyses were cross-validated across both platforms.

### 2.11. Subgroup and Sensitivity Analyses

Prespecified subgroup analyses were conducted by graft material type (e.g., xenograft, allograft, alloplast, autologous) and by healing duration (short-term ≤ 3 months vs. long-term > 3 months). Sensitivity analyses were performed by excluding high-risk-of-bias studies and outliers flagged by influence diagnostics.

### 2.12. Meta-Bias Assessment

Publication bias was evaluated through funnel plot inspection and Egger’s regression test, provided there were at least 10 studies for the outcome. For new bone formation, no evidence of publication bias was found (Egger’s *p* = 0.576).

### 2.13. Certainty of Evidence

The certainty of the evidence for each outcome was appraised using the GRADE approach, considering five domains: risk of bias, inconsistency, indirectness, imprecision, and publication bias. Each domain was independently assessed by two reviewers. The overall certainty for new bone formation was rated as moderate due to downgrades in risk of bias and inconsistency.

## 3. Results

### 3.1. Study Selection

The electronic search revealed 14.705 studies in total. Following screening of the article titles, 5.732 potentially relevant articles were identified after duplicate removal. Independent screening of abstracts resulted in the selection of 29 publications for possible inclusion. Following full-text screening, 22 of the 29 articles met the inclusion criteria and thus were included in the systematic review.

**Figure 1:**
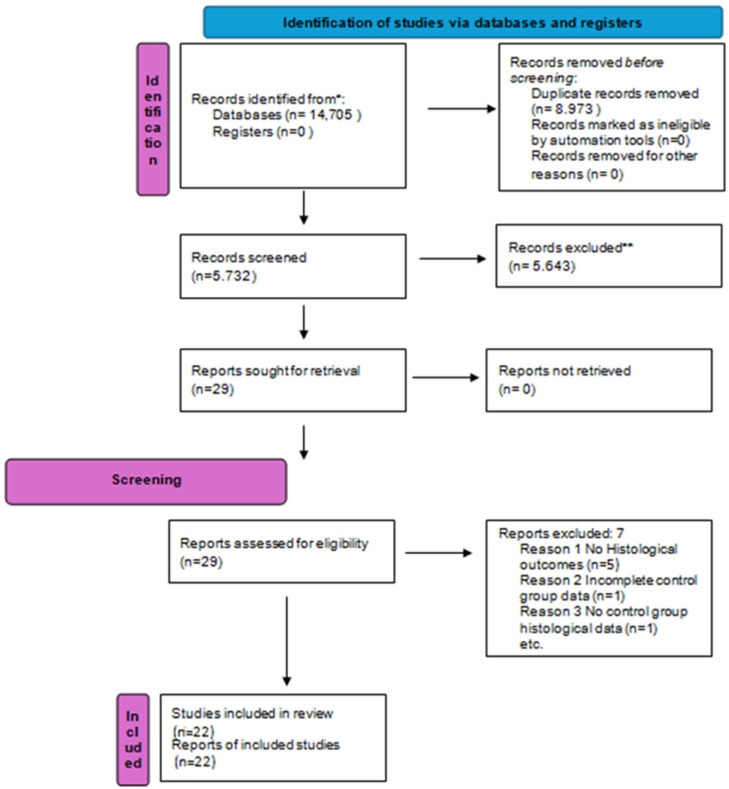
Flowchart summarizing the process of study identification, screening, eligibility assessment, and inclusion, in accordance with the PRISMA 2020 guidelines. * Records identified from database searches (e.g., PubMed, Scopus, Web of Science, Embase). No records were retrieved from study registries. ** Records excluded after title and abstract screening.

A total of 22 randomized controlled trials (RCTs) fulfilled the inclusion criteria and were incorporated into the quantitative synthesis. These studies collectively enrolled 505 patients allocated to socket preservation interventions and 311 patients managed with spontaneous healing. The study selection process, including identification, screening, and eligibility assessments, is detailed in Figure 1.

### 3.2. Study Characteristics

Table 1 summarizes the detailed characteristics of the included studies, including study design, type of grafting material, control interventions, sample sizes, follow-up durations, and histological assessment methods.

The present systematic review included 22 studies that evaluated histological outcomes following alveolar ridge preservation (ARP) using various grafting materials in comparison to spontaneous healing. All studies were randomized controlled trials (RCTs). The studies were conducted across diverse regions including Europe, Asia, North and South America, and the Middle East.

The sample sizes in intervention and control groups ranged from 5 to 60 subjects, with follow-up durations varying from 2 to 6 months post-extraction. Socket preservation procedures involved a broad spectrum of grafting materials such as platelet-rich fibrin (PRF), concentrated growth factors (CGFs), freeze-dried bone allograft (FDBA), demineralized FDBA (DFDBA), deproteinized bovine bone mineral with collagen (DBBM-C), and various synthetic alloplasts. In some cases, biologics such as Emdogain or bone marrow aspirate concentrate (BMAC) were added.

Histological assessment in all studies was performed using undecalcified sections, which were embedded in resin or plastic and processed for microscopic evaluation. Most studies applied toluidine blue, Hematoxylin and Eosin (H&E), or Masson’s trichrome stains to distinguish mineralized from soft tissue components. Histomorphometric outcomes typically included the percentage of newly formed bone, residual graft particles, and marrow or connective tissue, with quantitative analysis carried out using point-counting, image analysis software, or digital morphometry.

### 3.3. Risk of Bias Assessment

The methodological quality of the 22 included randomized controlled trials was evaluated using the Cochrane Risk of Bias 2.0 (RoB 2) tool, which assesses five domains: randomization process, deviations from intended interventions, missing outcome data, measurement of outcomes, and selection of the reported result. Overall, the quality of the studies was variable, with 10 trials judged to be at low risk of bias across all domains. These studies demonstrated clear randomization procedures, appropriate handling of interventions, minimal loss to follow-up, objective and standardized histological outcome measurements, and full protocol transparency.

Six studies were judged to raise some concerns regarding bias. These concerns were most frequently related to incomplete information on allocation concealment, possible deviations from the intended protocol, or insufficient clarity in outcome reporting. Although these studies did not exhibit high risk in any single domain, the lack of full methodological transparency warranted a conservative rating.

Five studies were considered to be at high risk of bias. Notably, studies such as Gabay (2022), Hauser (2013), Iasella (2003), and Ivanova (2021) [27,30,39] presented multiple sources of potential bias, including unclear or inappropriate randomization methods, high levels of missing data, and concerns about selective outcome reporting or outcome assessment blinding. These limitations could have introduced systematic errors in the estimation of treatment effects, particularly in studies relying on histological endpoints that are subject to variation based on sampling technique, region of interest, and examiner calibration.

The detailed assessments for each study are presented in Table 2. These risk of bias ratings were taken into account during sensitivity analyses and in the GRADE assessment of the overall certainty of the evidence. Notably, exclusion of high-risk studies in subgroup and sensitivity analyses did not materially alter the results’ direction or magnitude, supporting the findings’ robustness despite some methodological variability across the included trials.

### 3.4. Primary Outcome: New Bone Formation

#### 3.4.1. Overall Meta-Analysis

Table 3 presents the mean percentages, standard deviations, and sample sizes for new bone formation in both socket preservation and spontaneous healing groups across 22 randomized controlled trials. New bone formation ranged from 23.9% to 62.04% in the socket preservation groups, and from 5.98% to 39.69% in the spontaneous healing groups.

**Figure 2:**
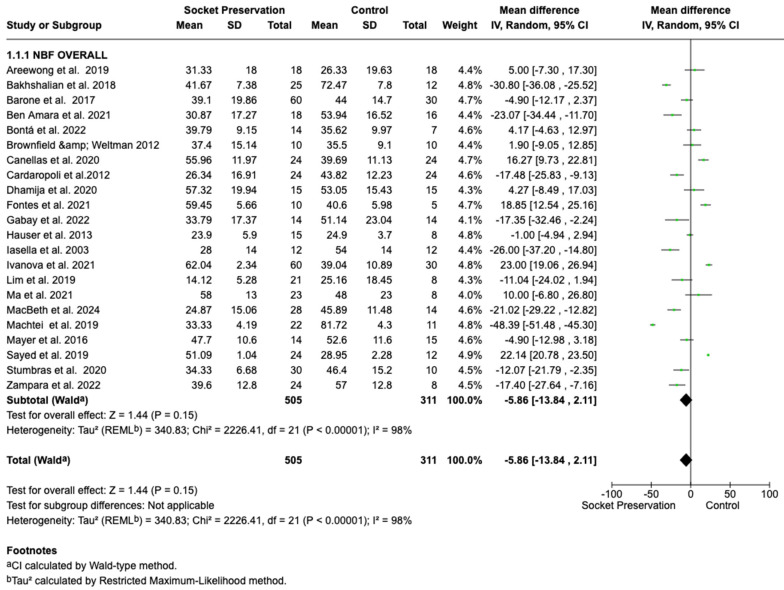
Forest plot of new bone formation (%) comparing socket preservation and spontaneous healing [17,18,19,20,21,22,23,24,25,26,27,28,29,30,31,32,33,34,35,36,37,38].

The primary meta-analysis, synthesizing data from all 22 studies, found no statistically significant difference in new bone formation between socket preservation and spontaneous healing. The pooled mean difference was −5.86% (95% confidence interval [CI]: −13.84% to 2.11%, *p* = 0.15), indicating a non-significant trend toward higher new bone formation in the control (spontaneous healing) group. However, the direction and magnitude of this effect should be interpreted with caution due to substantial heterogeneity (I^2^ = 98%), reflecting considerable variability in study protocols, materials, and healing times (Figure 2).

#### 3.4.2. Subgroup Analyses by Graft Material

Subgroup analyses were performed based on the type of graft material utilized. Socket preservation procedures involving autologous growth factors (PRF or PRP) demonstrated a statistically significant enhancement in new bone formation compared to spontaneous healing (mean difference = +16.28%; 95% CI: 7.53% to 25.03%; *p* < 0.0001), albeit with very high heterogeneity (I^2^ = 97%).

Xenografts without membrane application did not show a significant effect (mean difference = −25.52%; 95% CI: −97.60% to 46.56%; *p* = 0.27), whereas xenografts combined with collagen membranes produced a statistically significant reduction in new bone formation (mean difference = −22.47%; 95% CI: −33.59% to −11.34%; *p* = 0.003) with moderate heterogeneity (I^2^ = 57%).

Allografts and alloplasts demonstrated non-significant differences compared to spontaneous healing. Allografts yielded a pooled mean difference of −16.33% (95% CI: −47.90% to 15.24%; *p* = 0.20 and alloplasts yielded −14.76% (95% CI: −62.97% to 33.45%; *p* = 0.32), both with substantial heterogeneity.

**Figure 3:**
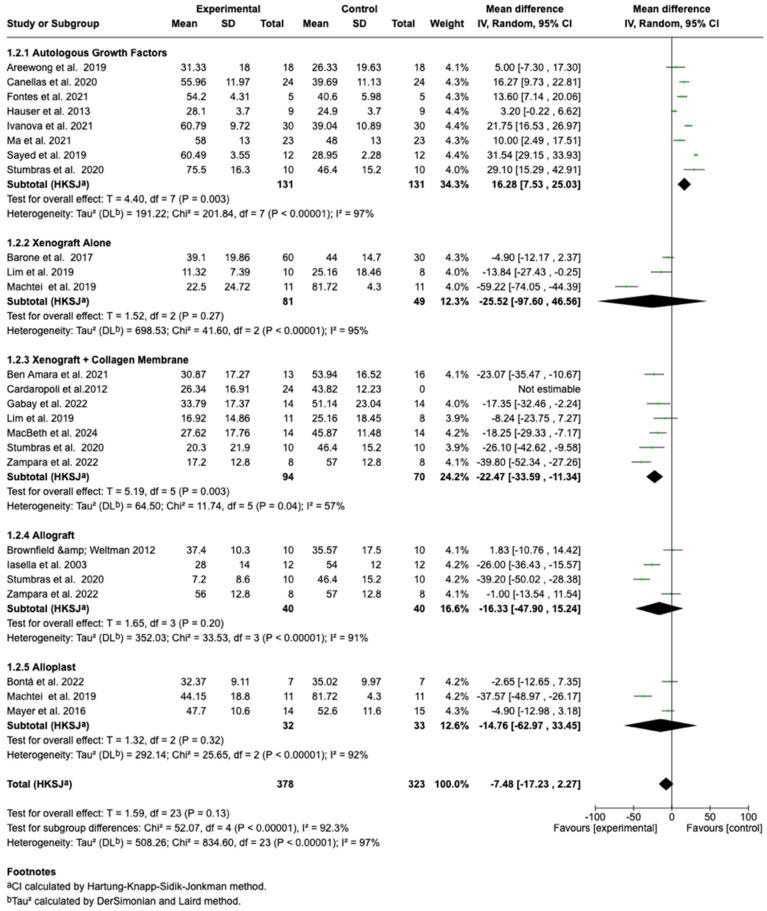
Forest plot displaying subgroup analyses of mean differences in new bone formation (%) between socket preservation and spontaneous healing, stratified by the type of grafting material used. Categories include autologous growth factors (e.g., PRF, PRP), xenografts with or without collagen membranes, allografts, and alloplasts [17,19,20,21,22,23,24,26,27,28,29,30,31,32,33,34,35,36,37,38].

A forest plot stratifying results by graft material category is presented in Figure 3.

A formal test for subgroup differences was statistically significant (Chi^2^ = 52.07, df = 4; *p < 0.00001*; I^2^ = 92.3%), indicating that graft material substantially influenced histological outcomes.

Following exclusion of studies judged to be at high risk of bias, results for autologous growth factors and xenografts combined with collagen membranes remained statistically significant. Autologous growth factors exhibited a mean difference of +16.28% (95% CI: 7.53% to 25.03%; *p < 0.0001*) with reduced heterogeneity (I^2^ = 65%), and xenografts with membranes showed a mean difference of −22.47%% (95% CI: −33.59%% to −11.34%%; *p = 0.04*) with moderate heterogeneity (I^2^ = 57%).

In contrast, results for xenografts without membranes, allografts, and alloplasts remained non-significant after excluding high-risk studies, although heterogeneity generally decreased.

#### 3.4.3. Subgroup Analysis by Healing Duration

A subgroup analysis based on healing duration was conducted, categorizing studies into short-term healing (≤3 months) and long-term healing (>3 months) groups. The pooled mean difference in the short-term healing subgroup was + 6.53% (95% CI: −1.57%% to 14.64%; *p* = 0.11), with substantial heterogeneity (I^2^ = 87%). The mean difference in the long-term healing subgroup was −10.55% (95% CI: −20.27% to −0.83%%; *p* = 0.03), again accompanied by extremely high heterogeneity (I^2^ = 98%).

**Figure 4:**
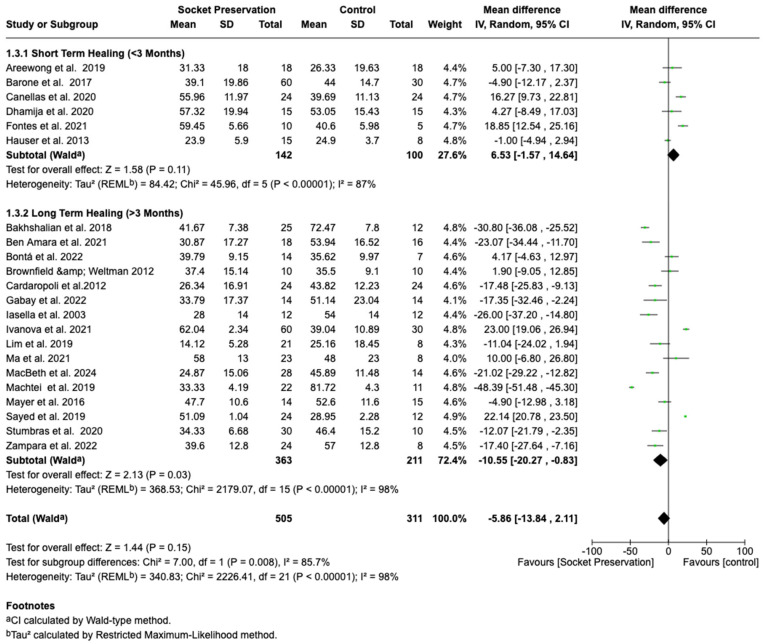
Forest plot illustrating subgroup analyses of mean differences in new bone formation (%) between socket preservation and spontaneous healing, categorized by healing duration. Studies were grouped into short-term healing (≤3 months) and long-term healing (>3 months) [17,18,19,20,21,22,23,24,25,26,27,28,29,30,31,32,33,34,35,36,37,38].

After sensitivity analyses excluding outliers, heterogeneity among short-term studies decreased to I^2^ = 0%, and a consistent but non-significant result favoring socket preservation persisted. Conversely, in the long-term healing group, a statistically significant benefit favoring socket preservation emerged (mean difference = −16.30%; 95% CI: −21.61% to −10.98%; *p* < 0.00001), with moderate residual heterogeneity (I^2^ = 51%). A forest plot stratifying results by healing duration is presented in Figure 4.

#### 3.4.4. Assessment of Publication Bias

Publication bias was evaluated through both visual and statistical approaches. Funnel plot inspection revealed a reasonably symmetrical distribution of effect sizes around the pooled mean, with no clear evidence of small-study effects. To statistically confirm this observation, Egger’s regression test was conducted. The intercept was not significantly different from zero (*p* = 0.576), indicating that the magnitude of the treatment effect was not associated with study precision. Therefore, there is no statistical evidence of publication bias affecting the analysis of new bone formation.

The lack of funnel plot asymmetry and a non-significant Egger’s test suggest that the meta-analysis is not substantially influenced by selective reporting of favorable results, strengthening the reliability of the overall conclusions.

#### 3.4.5. Meta-Regression Analysis

A weighted meta-regression was conducted to evaluate whether two prespecified clinical moderators—membrane application (binary: yes/no) and healing duration (continuous, in months)—could explain heterogeneity in the reported mean differences in new bone formation.

The model did not reach statistical significance (F(2, 18) = 0.56; *p* = 0.583) and exhibited a low explanatory power (R^2^ = 5.8%). Individual regression coefficients showed the following patterns:

Membrane application positively affected new bone formation (β = +15.37%), suggesting that barrier use might enhance graft stabilization and bone regeneration. However, this trend did not reach statistical significance (*p* = 0.489), and the 95% confidence interval crossed zero.

Healing duration was negatively associated with new bone formation (β = −5.51% per month; *p* = 0.332), implying that longer healing times may lead to remodeling or resorption dynamics that reduce measurable new bone percentage. Again, this trend was not statistically significant.

Although the meta-regression did not reach statistical significance (*p* = 0.583), this is likely due to the dominant influence of graft material type, which may have overshadowed the smaller effects of healing duration and membrane application. The variability in biomaterial behavior—particularly between slow- and fast-resorbing grafts—may explain why these secondary moderators failed to demonstrate independent significance.

### 3.5. Residual Graft Material

#### 3.5.1. Meta-Analysis of Residual Graft Percentages

A total of 17 study arms from randomized controlled trials (RCTs) reported quantitative histomorphometric data on residual graft material following alveolar ridge preservation (ARP) procedures (Table 4). These study arms—some of which originate from multi-arm RCTs—differ in graft material type and membrane application but all provided relevant mean values and standard deviations. The pooled mean residual graft content was 20.49% (95% confidence interval [CI]: 16.78% to 24.20%), reflecting the average proportion of the post-extraction socket area occupied by remaining biomaterial at the time of biopsy. The corresponding 95% prediction interval ranged widely from 6.58% to 34.40%, indicating that in future settings, residual graft content could fall anywhere within this range depending on patient- and protocol-specific factors.

**Figure 5:**
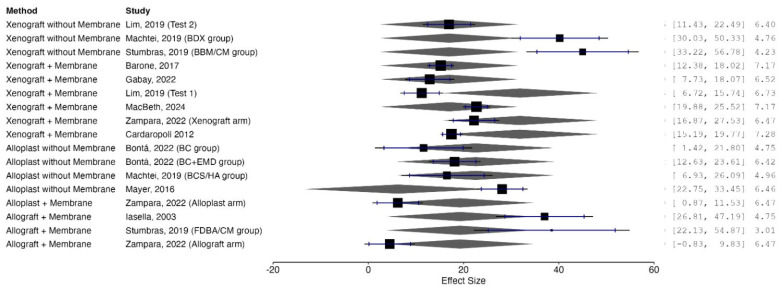
Forest plot of residual graft material (%) including pooled mean estimate (20.49%, 95% CI 16.78–24.20%) and heterogeneity metrics (I2 = 88.99%, τ2 = 46.78, τ = 6.84) [19,21,24,27,29,31,33,34,35,37,38].

Statistical heterogeneity was substantial (I^2^ = 88.99%, τ^2^ = 46.78, τ = 6.84), supporting the use of a random-effects model. Residual heterogeneity remained significant (Q_e_(12) = 109.01, *p* < 0.001). A forest plot summarizing the residual material values by study is shown in Figure 5.

#### 3.5.2. Subgroup and Meta-Regression Analysis

A meta-regression analysis was conducted with graft material category (xenograft, allograft, alloplast) and membrane application (yes/no) as moderators to explore sources of heterogeneity. The omnibus test approached statistical significance (Q_m_(4) = 11.04, *p* = 0.026), suggesting a potential but inconclusive contribution of these moderators to the observed variability.

Although none of the individual moderator coefficients reached statistical significance (all *p* > 0.05), clinically meaningful trends were observed:

Alloplasts with membrane were associated with the lowest residual material levels (β = −16.34%, 95% CI: −33.74% to 1.06%; *p* = 0.066), indicating enhanced biomaterial turnover.

Xenografts without membrane showed higher residual content (β = +9.31%, 95% CI: −4.19% to 22.80%; *p* = 0.177), consistent with their well-documented slow resorption profile.

Xenografts with membrane showed a modest reduction (β = −5.55%, 95% CI: −16.83% to 5.73%; *p* = 0.335), possibly due to improved containment and integration.

Allograft + Membrane was used as the reference category (β = 22.54%, 95% CI: 12.81% to 32.27%; *p* < 0.001), while Alloplast without Membrane demonstrated a small, non-significant decrease (β = −3.28%, 95% CI: −15.70% to 9.14%; *p* = 0.604) 

The proportion of between-study variance explained by these moderators (pseudo-R^2^ ≈ 5.4%) suggests that graft type and membrane usage account for a small but meaningful portion of the heterogeneity. Other likely contributing factors include biopsy timing, socket morphology, flap design, and inter-patient variability.

#### 3.5.3. Sensitivity Analyses and Influential Studies

Influence diagnostics, including DFFITS and Cook’s Distance, identified several studies with potential outlier behavior—most notably Lim et al., 2019 (Test 2) and Zampara et al., 2022 (allograft arm) [31,38]. Upon exclusion of these studies in a sensitivity analysis, model direction and magnitude remained largely unchanged, indicating that the observed patterns were robust to the influence of outliers.

These findings support the hypothesis that xenografts, especially when used without membrane, are associated with higher persistence of residual material within the socket. This is consistent with their slow resorption kinetics and common histological profiles. In contrast, synthetic alloplasts exhibit lower residual volumes, particularly when combined with membranes, suggesting a more rapid turnover and replacement by vital bone. Although these trends did not achieve statistical significance, they are clinically relevant and consistent with the biological behavior of the materials. High residual heterogeneity underscores the need for standardized histological protocols and longer follow-up durations in future trials. This finding indicates that healing time may partly influence the histological outcomes, although high residual heterogeneity persists within both subgroups.

### 3.6. GRADE Assessment: Certainty of Evidence for New Bone Formation

The certainty of evidence for the overall effect of socket preservation on new bone formation was assessed using the GRADE (Grading of Recommendations Assessment, Development, and Evaluation) framework. The evaluation considered methodological quality, consistency of results, directness of evidence, precision of effect estimates, and potential publication bias.

Based on this structured appraisal, the certainty of evidence was rated as moderate. This rating reflects downgrading due to serious risk of bias and inconsistency, despite no concerns for indirectness or publication bias, and only moderate concerns regarding precision. The results are summarized in Table 5.

## 4. Discussion

The present systematic review and meta-analysis investigated the histological outcomes of alveolar ridge preservation (ARP) compared to spontaneous healing following tooth extraction. The primary outcome was new bone formation, while secondary outcomes included residual graft material and connective tissue content. Although ARP has become a widely adopted procedure in implant dentistry to limit post-extraction dimensional changes, its histological efficacy remains a subject of ongoing debate. This study, synthesizing data from 22 randomized controlled trials, aimed to provide clarity regarding the regenerative potential of various ARP strategies.

### 4.1. Summary of Principal Findings

The meta-analysis found no statistically significant overall difference in new bone formation between ARP and spontaneous healing. The pooled mean difference was −5.86% (95% CI: −13.84% to 2.11%, *p* = 0.15), favoring spontaneous healing, albeit not significantly. This observation aligns with findings from Adams et al. (2022), who reported that many bone substitute materials, particularly xenografts, may delay the healing process and yield lower proportions of newly formed bone compared to unassisted healing [40]. They further argued that no grafting material has shown superior histological bone formation compared to extraction alone in high-quality trials.

Despite the lack of an overall benefit, subgroup analyses demonstrated that autologous platelet concentrates (e.g., platelet-rich fibrin [PRF] or concentrated growth factors [CGFs]) significantly improved new bone formation relative to controls (mean difference = + 16.28%, 95% CI: 7.53% to 25.03%; *p* < 0.0001). This finding is consistent with clinical trials and reviews that highlight the regenerative potential of autologous biologics in promoting angiogenesis and osteoblastic activity through the sustained release of growth factors [40]. In contrast, xenografts without membrane coverage did not yield significant improvements, whereas xenografts combined with collagen membranes were associated with significantly higher new bone formation (mean difference = −22.47%, 95% CI: −33.59% to −11.34%; *p* = 0.04), corroborating earlier systematic reviews by Canullo et al. (2022) and Avila-Ortiz et al. [9,15].

Importantly, studies that employed slow-resorbing xenografts or alloplasts frequently demonstrated substantial amounts of residual graft material, with pooled mean residual contents around 20.49%. These outcomes highlight the biological trade-off between dimensional stability and bone turnover, as persistent biomaterial may maintain ridge volume but limit the proportion of vital bone. Histomorphometric studies, such as those by Mardas et al. (2023) and Machtei et al. (2019), have previously noted that slow-resorbing biomaterials like deproteinized bovine bone mineral (DBBM) may persist within the socket and reduce the proportion of vital bone even after several months of healing [10,34]. This raises concerns about potential implications for future implant osseointegration.

Furthermore, evidence from animal and human models has suggested that the natural healing of extraction sockets—when uninterrupted by foreign graft material—can result in substantial spontaneous bone regeneration [41]. Their cross-sectional clinical data showed that non-grafted sockets consistently achieved adequate bucco-lingual bone width for implant placement, even in the presence of minor dehiscence, challenging the necessity of routine grafting. Similarly, Ram et al. (2023) demonstrated comparable histological and molecular bone remodeling responses in grafted versus non-grafted extraction sockets, emphasizing the inherent osteogenic capacity of the alveolus [42].

Taken together, the current findings suggest that while ARP can provide structural benefits and enhance soft tissue contour, its superiority in terms of vital bone formation is not universal and appears highly dependent on material type, membrane use, and healing time.

### 4.2. Biological and Clinical Interpretation

#### 4.2.1. New Bone Formation

Paradoxically, several histological studies report that spontaneous healing may result in equal or even superior percentages of vital bone compared to socket preservation using xenografts. For instance, Katorza et al. (2022) reported 51.1% ± 23.0% new bone in naturally healed sockets versus 33.8% ± 17.4% in DBBM-C-treated sites, despite better volumetric maintenance in the latter [43]. This paradox reflects the distinction between *radiographic bone fill* and *histological bone quality*. Although biomaterial-filled sockets may appear denser in radiographic imaging, histological sections often reveal that a substantial proportion of the radiopacity is due to residual graft particles rather than newly formed bone [27].

This discrepancy underscores the importance of differentiating between structural volume and true osseous regeneration. DBBM, a slowly resorbing xenograft, tends to persist long-term, acting more as a scaffold than a source of vital bone, which may compromise early bone remodeling dynamics [27,44]. Natural healing, in contrast, leverages the body’s intrinsic remodeling potential to produce a higher fraction of vital bone in the early months post-extraction.

The role of autologous platelet concentrates such as platelet-rich fibrin (PRF) and concentrated growth factors (CGFs) further illustrates this principle. These materials stimulate osteogenesis through a fibrin matrix rich in cytokines and growth factors like TGF-β and PDGF, which promote early angiogenesis and recruitment of osteoprogenitor cells [45,46]. In the studies using PRF, healing times as short as 4–8 weeks already yielded enhanced tissue maturation compared to controls.

#### 4.2.2. Residual Graft Material

Residual graft presence is directly related to material composition and resorption kinetics.

Studies consistently show that DBBM demonstrates high persistence rates, with residual particle percentages commonly between 15% and 19.3% after 6–9 months. This finding aligns with our subgroup data (Table 4), where xenografts—particularly those used without membranes—tended to yield lower proportions of vital bone compared to natural healing (MD = −25.52, 95%CI −97.60 to 46.45; *p* = 0.27), consistent with their slow resorption profile and long-term material persistence. This pattern supports the interpretation that xenografts primarily act as space-maintaining scaffolds rather than active osteoconductive matrices [27,43]. This contrasts sharply with natural healing, where no exogenous material remains, and all bone observed is vital.

Alloplastic materials such as β-TCP or biphasic calcium phosphate demonstrate more variable resorption. In some cases, these materials support faster turnover but may also provoke inconsistent remodeling outcomes depending on particle size and porosity [47].

Clinically, while residual DBBM does not appear to negatively affect implant primary stability its presence can complicate future re-entry surgeries and may limit complete osseointegration at the graft–host interface [39,43,48]. Therefore, the choice of grafting material must weigh volumetric stability against long-term remodeling efficiency.

#### 4.2.3. Membrane Use

The addition of membranes—particularly in xenograft-based preservation—was associated with slightly more favorable and consistent histological outcomes (MD = −22.47, 95% [−33.59, −11.34], *p* = 0.003; I^2^ = 57%). In the study by Gabay et al. (2022), DBBM-C combined with collagen membrane (CMXS) resulted in stabilized clot architecture and reduced soft tissue ingrowth, facilitating favorable histological conditions despite lower new bone percentages than spontaneous healing [27]. These findings align with other reports showing that membranes can enhance soft tissue healing and potentially increase the proportion of keratinized tissue [49,50].

Zhao et al. (2020) also concluded that the choice of membrane (collagen vs. PTFE) had no statistically significant impact on bone regeneration outcomes, suggesting that the biological advantage of membranes may be more pronounced in tissue handling and clinical management than in the final percentage of new bone [44].

#### 4.2.4. Healing Time

Healing duration plays a pivotal role in the interpretation of histological findings. Short-term biopsies (e.g., 3–4 months) may capture early scaffold incorporation but underestimate later remodeling. Studies included in this review indicate a trend toward increased new bone formation percentages with longer healing periods (over 3 months) compard to short term (MD = −10.55, 95% CI [−20.27, −0.83], *p* = 0.03). This observation indicates that true osseous remodeling may become more apparent after prolonged healing periods, while short-term biopsies could primarily reflect residual graft material and thus underestimate the extent of mature bone [43,44]. Histological biopsy remains the gold standard for assessing true osteogenesis. This raises a clinical caution: radiographic bone gain does not necessarily equate to functional bone maturity, especially in grafted sites. The delayed emergence of statistically significant benefits beyond three months likely reflects the integration kinetics of slow-resorbing materials such as DBBM. Early healing phases are dominated by scaffold incorporation, whereas true bone remodeling and mineral maturation become evident only after 4–6 months.

### 4.3. Comparison to Previous Literature

The findings of this meta-analysis are consistent with those of Avila-Ortiz et al. (2019) and Vignoletti et al. (2012), who demonstrated that alveolar ridge preservation (ARP) reduces dimensional changes compared to unassisted healing. However, this review goes further by focusing exclusively on randomized controlled trials (RCTs) with histological endpoints, thereby enhancing the level of evidence and minimizing [8,15].

Unlike previous meta-analyses, which often pooled heterogeneous study designs or relied on radiographic surrogate endpoints, this study distinguishes between volumetric preservation and biological bone regeneration. Meta-regression and subgroup analyses further allowed for quantification of the effects of graft type, membrane use, and healing duration on the histological composition of the regenerated tissue.

Overall, while ARP—particularly when using xenografts with or without collagen membranes—appears to preserve ridge dimensions radiographically, the pooled histological analysis did not demonstrate a statistically significant advantage in new bone formation over spontaneous healing (MD = −5.86, 95% CI [ −13.84, 2.11], *p* = 0.15). These results suggest that radiographic stability does not necessarily translate into histological superiority. This distinction is crucial when planning for implant placement, particularly in esthetic zones, where soft tissue integration and osseointegration are equally critical.

### 4.4. Methodological Strengths

This systematic review and meta-analysis adhered strictly to the PRISMA 2020 guidelines, ensuring transparency in reporting and methodological rigor. The protocol was prospectively registered in the PROSPERO database (CRD420251019531), predefining objectives, eligibility criteria, and outcomes. Only randomized controlled trials (RCTs) were included, minimizing selection bias and enhancing the reliability of the synthesized estimates. A comprehensive range of statistical procedures—including subgroup analyses by graft type and healing time, sensitivity analyses excluding high-risk studies, and random-effects meta-regression—allowed for exploration of potential sources of heterogeneity. Moreover, the certainty of evidence for each outcome was evaluated using the GRADE framework, offering a structured appraisal of confidence in effect estimates based on risk of bias, inconsistency, indirectness, imprecision, and publication bias.

### 4.5. Limitations

Despite the strengths of this analysis, several limitations must be acknowledged. First, high statistical heterogeneity persisted across most outcomes, even after stratification and sensitivity testing. This heterogeneity likely stems from biological and methodological variability among studies, including differences in biopsy timing (ranging from 2 to 9 months), sampling location (central vs. marginal socket areas), and histological processing (e.g., undecalcified vs. decalcified sections; staining methods such as toluidine blue, H&E, or trichrome). This high degree of heterogeneity underscores the urgent need for methodological standardization. Future studies should clearly specify biopsy sites (central vs. marginal), unify staining protocols, and consistently report bone density or mineralization indices to distinguish immature from mature bone tissue. Furthermore, reporting standards varied widely, with some trials omitting key parameters such as residual graft percentage or graft material density. Although only RCTs were included, not all demonstrated low risk of bias; approximately one-third of included studies were rated as having some concerns or high risk of bias due to unclear randomization procedures, missing data, or non-blinded outcome assessments. Lastly, most studies had relatively short follow-up durations (≤6 months), limiting conclusions regarding the long-term biological fate of grafted materials and their integration. Given the very high heterogeneity (I^2^ = 98%) in the overall meta-analysis, the pooled estimate should be interpreted with caution. Greater weight should be placed on subgroup analyses stratified by graft material, where more clinically meaningful and statistically reliable trends were observed.

### 4.6. Clinical Implications

Subgroup analyses indicated that histological outcomes were primarily driven by the biological nature of the material—its resorption rate and osteoinductive capacity—rather than by procedural variables. This suggests that material-related factors, including resorption kinetics and biological activity, overshadowed procedural variables in explaining between-study variance.

Moreover, the trend toward improved outcomes at longer healing times, reaching statistical significance only after sensitivity analysis, may reflect the gradual integration of slowly resorbing biomaterials such as xenografts. While these materials provide mechanical stability and space maintenance, their limited early resorption may delay full osseous substitution. Consequently, early histological samples (<3 months) may underestimate the eventual regenerative potential of such biomaterials.

From a clinical standpoint, these results underscore the importance of tailoring graft selection to individual patient needs. In cases where ridge contour maintenance and esthetic outcomes are prioritized, the use of slower-resorbing xenografts may still be acceptable—even if histologically they exhibit lower proportions of vital bone—since they maintain volume and support implant placement with predictable stability. Conversely, autologous concentrates like PRF or CGF may be preferable when faster osseointegration and reduced residual material are desired. Economic and patient-related factors, including cost, morbidity, and postoperative comfort, should also guide decision-making. Ultimately, histological performance must be weighed against overall clinical efficiency, esthetic demands, and long-term implant success.

### 4.7. Recommendations for Future Research

Future studies should adopt standardized histological protocols with consistent timing of biopsies (e.g., 3, 6, and 9 months), unified staining techniques, and calibrated histomorphometric measurements. Large-scale, multicenter RCTs with extended follow-up (≥12 months) are needed to evaluate long-term implant success, osseointegration, and the remodeling dynamics of different grafting materials. Comparative trials between autologous biologics and conventional grafts will be particularly valuable in elucidating the relative merits of bioactive versus passive scaffolds. Moreover, future research should integrate cost-effectiveness analyses and patient-reported outcomes—including pain, morbidity, and esthetic satisfaction—to inform treatment selection beyond histological endpoints.

## 5. Conclusions

In conclusion, while socket preservation strategies are effective in maintaining alveolar ridge dimensions and may benefit future implant placement, their histological superiority over spontaneous healing remains inconsistent and material-dependent. Radiographic or volumetric gains do not necessarily correlate with increased vital bone content. The integration of biologically active and resorbable materials, such as PRF and collagen matrices, combined with proper soft tissue management (e.g., membrane use), appears to offer the most favorable regenerative profile. These insights underscore the need for individualized treatment planning grounded in both clinical outcomes and biological integration. Despite its well-documented benefit in preserving alveolar dimensions, ARP may not necessarily enhance vital bone formation compared to spontaneous healing. This paradox underscores the biological trade-off between structural stability and true histological regeneration.

## 6. Registration and Protocol

This systematic review was prospectively registered in the International Prospective Register of Systematic Reviews (PROSPERO) under the registration number CRD420251019531. The full review protocol, including the PICOS framework and prespecified analysis plan, is publicly accessible via the PROSPERO registry at: https://www.crd.york.ac.uk/prospero/display_record.php?ID=CRD420251019531. Date of registration in PROSPERO: 26 March 2025.

## Figures and Tables

**Table 1:** Study characteristics of included trials evaluating histological outcomes following socket preservation [17,18,19,20,21,22,23,24,25,26,27,28,29,30,31,32,33,34,35,36,37,38].

Study (Author, Year)	Country	Study Design	Grafting Material	Control	Sample Size (Intervention/Control)	Follow-Up (Months)	Histological Assessment Method
Areewong, 2019 [17]	Thailand	RCT	PRF	Spontaneous healing	18/18	2	Undecalcified sections; toluidine blue staining; histomorphometry under light microscopy
Bakhshalian, 2018 [18]	USA	RCT	SocketKAP/SocketKAP + Xenograft/SocketKAGE + Xenograft + SocketKAP	Spontaneous healing	6/11/3/2	6	Ground section histomorphometry; specific staining method not reported
Barone, 2017 [19]	Italy	RCT	Xenograft	Spontaneous healing	30/30/30	3	Undecalcified sections; histomorphometry of new bone, residual graft, and connective tissue
BenAmara, 2021 [20]	Tunisia	RCT	DBBM-C + collagen membrane	Spontaneous healing	18/16	6	Undecalcified sections; light microscopy with morphometric analysis
Bontá, 2022 [21]	Argentina	RCT	Alloplast/Alloplast + Emdogain	Spontaneous healing	7/7/7	6	H&E and Masson’s trichrome-stained sections; histomorphometry
Brownfield&Weltman, 2012 [22]	USA	RCT	FDBA	Spontaneous healing	10/10	2.5–3	Undecalcified histological sections; staining method not specified
Canellas, 2020 [23]	Brazil	RCT	PRF	Spontaneous healing	2184/24	3	Resin-embedded undecalcified sections; toluidine blue; image analysis
Cardaropoli,2012 [24]	Italy	RCT	Bovine bone mineral (Bio-Oss Collagen) + Porcine collagen membrane (Bio-Gide)	Spontaneous healing	24/24 (48 sockets from 41 patients)	4	Undecalcified resin-embedded sections; Azure II and pararosaniline staining; histomorphometric analysis under light microscopy)
Dhamija, 2020 [25]	India	RCT	FDBA + PRF	Spontaneous healing	15/15	3–4	Toluidine blue-stained undecalcified sections; histomorphometry
FontesMartins, 2020 [26]	Brazil	RCT	PRF/PRF + BMAC	Spontaneous healing	5/5/5	6	Undecalcified histological sections; method of staining not described
Gabay, 2022 [27]	Israel	RCT	Xenograft (DBBM-C) + collagen membrane	Spontaneous healing	14/14	6	Masson’s trichrome staining of undecalcified sections; quantitative light microscopy
Hauser, 2013 [28]	Switzerland	RCT	PRF/PRF-Flap	Spontaneous healing	9/6/8	2	Semithin ground sections stained with toluidine blue; light microscopy
Iasella, 2003 [29]	USA	RCT	FDBA + collagen membrane	Spontaneous healing	12/12	6	Undecalcified histological sections; morphometric software analysis
Ivanova, 2021 [30]	Bulgaria	RCT	PRF + Allograft/PRF	Spontaneous healing	30/30/30	4	Histomorphometry; specific details of staining not reported
Lim, 2019 [31]	South Korea	RCT	DBBM-C + collagen membrane/DBBM-C	Spontaneous healing	11/10/8	4	Undecalcified sectioning; digital image analysis
Ma, 2021 [32]	China	RCT	CGFs (Concentrated Growth Factors)	Spontaneous healing	23/23	3.5	Histology and histomorphometry of new bone vs. residual graft; image analysis tools
MacBeth, 2024 [33]	UK	RCT	Xenograft	Spontaneous healing	30/30	4	Resin-embedded sections; toluidine blue staining; quantitative histomorphometry
Machtei, 2019 [34]	Israel	RCT	DBBM vs. Alloplast	Spontaneous healing	11/11/11	4–4.5	Undecalcified ground sections; standardized light microscopy
Mayer, 2016 [35]	USA	RCT	Alloplast	Spontaneous healing	14/15	5	H&E-stained undecalcified sections; point-counting histomorphometry
Sayed, 2019 [36]	Egypt	RCT	PRF/PRF + Alloplast	Spontaneous healing	12/12/12	3	Plastic-embedded sections; toluidine blue; histomorphometric measurement
Stumbras, 2019 [37]	Lithuania	RCT	Xenograft/Allograft/PRGF	Spontaneous healing	10/10/10/10	3	Undecalcified resin blocks; toluidine blue staining; light microscopy
Zampara, 2022 [38]	Greece	RCT	Allograft/Xenograft/Alloplast	Spontaneous healing	8/8/8/8	6	Light microscopy on stained undecalcified sections; histomorphometry

RCT, randomized controlled trial, PRF, platelet-rich fibrin, CGFs, concentrated growth factors, FDBA, freeze-dried bone allograft, demineralized freeze-dried bone allograft, DBBM, deproteinized bovine bone mineral, DBBM-C, deproteinized bovine bone mineral with collagen, BMAC, bone marrow aspirate concentrate, H&E, hematoxylin and eosin, KAP, keratinized autologous punch, KAGE, keratinized autologous graft extension.

**Table 2:** Risk of bias assessment of included RCTs (Cochrane RoB 2 Tool) [17,18,19,20,21,22,23,24,25,26,27,28,29,30,31,32,33,34,35,36,37,38].

Study (Author, Year)	Domain 1: Randomization	Domain 2: Deviations from Intervention	Domain 3: Missing Data	Domain 4: Outcome Measurement	Domain 5: Selective Reporting	Overall Risk of Bias
Areewong, 2019 [17]	Low	Low	Low	Low	Some concerns	Low
Bakhshalian, 2018 [18]	Low	Low	Some concerns	Low	Low	Low
Barone, 2017 [19]	Low	Low	Low	Low	Low	Low
Ben Amara, 2021 [20]	Low	Low	Some concerns	Low	Low	Low
Bontá, 2022 [21]	Some concerns	Low	Low	Some concerns	Low	Some concerns
Brownfield&Weltman, 2012 [22]	Low	Low	Low	Low	Low	Low
Canellas,2020 [23]	Low	Low	Low	Low	Low	Low
Cardaropoli,2012 [24]	Low	Low	Low	Low	Low	Low
Dhamija, 2020 [25]	Some concerns	Low	Low	Low	Some concerns	Some concerns
Fontes Martins, 2021 [26]	Low	Low	Low	Low	Some concerns	Low
Gabay, 2022 [27]	High	Some concerns	Low	Some concerns	High	High
Hauser, 2013 [28]	High	Low	Some concerns	Low	Some concerns	High
Iasella, 2003 [29]	Some concerns	High	Some concerns	High	High	High
Ivanova, 2021 [30]	High	Low	Low	Low	Some concerns	High
Lim, 2019 [31]	Low	Low	Low	Low	Low	Low
Ma, 2021 [32]	Low	Low	Low	Low	Low	Low
MacBeth, 2024 [33]	Low	Low	Low	Low	Low	Low
Machtei, 2019 [34]	Low	Low	Low	Low	Low	Low
Mayer, 2016 [35]	Some concerns	Low	Low	Low	Some concerns	Some concerns
Sayed, 2019 [36]	Some concerns	Some concerns	Low	Some concerns	Low	Some concerns
Stumbras, 2020 [37]	Low	Low	Some concerns	Low	Some concerns	Some concerns
Zampara, 2022 [38]	Low	Low	Low	Low	Low	Low

**Table 3:** Summary of mean new bone formation (%) as assessed histomorphometrically in included randomized controlled trials comparing socket preservation with spontaneous healing. The table reports the mean percentage of newly formed bone, standard deviation (SD), and sample size (N) for both the socket preservation (intervention) and spontaneous healing (control) groups [17,18,19,20,21,22,23,24,25,26,27,28,29,30,31,32,33,34,35,36,37,38].

Study (Author, Year)	Socket Preservation Mean (%)	Socket Preservation SD (%)	Socket Preservation N	Control Mean (%)	Control SD (%)	Control N
Areewong et al., 2019 [17]	31.33	18	18	26.33	19.63	18
Bakhshalian et al., 2018 [18]	41.67	7.38	12	27.47	7.8	12
Barone et al., 2017 [19]	41.64	19.86	44	35.9	14.7	30
Ben Amaral et al., 2021 [20]	30.87	17.27	16	15.62	15.62	16
Bontà et al., 2022 [21]	39.79	9.97	14	35.62	9.97	14
Cardaropoli et al., 2012 [24]	26.34	16.91	24	43.82	12.23	24
Brownfield & Weltman, 2012 [22]	47.14	9.1	10	35.9	9.1	10
Canellas et al., 2020 [23]	55.66	11.13	24	39.89	11.13	24
Dhamija et al., 2020 [25]	57.32	9.34	15	15.43	15.43	15
Fontes et al., 2021 [26]	49.5	5.96	8	5.98	5.98	8
Gaby et al., 2022 [27]	39.7	17.37	14	23.04	23.04	14
Hauser et al., 2013 [28]	23.9	5.7	15	24.9	3.7	15
Iasella et al., 2003 [29]	62.04	2.34	30	39.04	10.89	30
Ivanova et al., 2021 [30]	39.6	9.34	18	10.89	10.89	18
Lim et al., 2019 [31]	14.12	1.9	18	18.45	18.45	18
Ma et al., 2021 [32]	5.8	13	23	48	23	23
Macbeth et al., 2024 [33]	24.87	15.06	22	45.89	11.48	23
Machetti et al., 2019 [34]	33.33	4.36	26	81.72	4.3	26
Mayer et al., 2018 [35]	37.6	6.47	14	29.85	2.28	12
Sayed et al., 2018 [36]	51.0	9.04	10	29.85	2.28	12
Stumbras et al., 2020 [37]	34.33	5.88	40	48.4	15.2	40
Zamparra et al., 2022 [38]	39.6	12.8	8	57.0	12.8	8

**Table 4:** Residual graft material across included study arms [19,21,24,25,27,29,31,33,34,35,37,38].

Category	Study (Author, Year)	Graft Material	Membrane	Healing	Residual Graft (%) < br > (Mean ± SD)	Sample Size (*n*)
Xenograft + Membrane	Barone, 2017 [19]	Porcine xenograft	Yes	Open	15.2% ± 7.87%	30
	Cardaropoli, 2012 [24]	DBBM-C	Yes	Open	18.46 ± 11.18%	24
	Gabay, 2022 [27]	DBBM-C	Yes	Open	12.9% ± 9.88%	14
	Lim, 2019 (Test 1) [31]	DBBM-C + NBCM	Yes	Open	11.23% ± 7.64%	11
	MacBeth, 2024 [33]	DBBM-C	Yes	Open	22.7% ± 7.9%	30
	Zampara, 2022 (Xenograft arm) [38]	DBBM	Yes	Open	22.2% ± 7.7%	8
Xenograft without Membrane	Lim, 2019 (Test 2) [31]	DBBM-C	No	Open	16.96% ± 8.93%	10
	Machtei, 2019 (BDX group) [33]	Bio-Oss	No	Open	40.18% ± 17.2%	11
	Stumbras, 2019 (BBM/CM group) [37]	BBM + Collagen membrane	No (exposed)	Open	45.0% ± 19.0%	10
Allograft + Membrane	Iasella, 2003 [29]	FDBA	Yes	Open	37% ± 18%	12
	Stumbras, 2019 (FDBA/CM group) [37]	FDBA + Collagen membrane	Yes	Open	38.5% ± 26.4%	10
	Zampara, 2022 (Allograft arm) [38]	Human cancellous allograft	Yes	Open	4.5% ± 7.7%	8
Allograft without Membrane	Dhamija, 2020 [25]	DFDBA + PRF	No	Primary closure	1.5% (SD not reported)	15
Alloplast + Membrane	Zampara, 2022 (Alloplast arm) [38]	Alloplast (Bondbone)	Yes	Open	6.2% ± 7.7%	8
Alloplast without Membrane	Bontá, 2022 (BC group) [21]	Alloplast	No	Open	11.61% ± 13.75%	7
	Bontá, 2022 (BC + EMD group) [21]	Alloplast + EMD	No	Open	18.12% ± 7.42%	7
	Machtei, 2019 (BCS/HA group) [34]	BCS/HA	No	Open	16.51% ± 16.2%	11
	Mayer, 2016 [35]	Alloplast	No	Open	28.1% ± 10.2%	14

**Table 5:** GRADE summary of findings—new bone formation.

Grade Domain	Judgment	Explanation
Risk of bias	⬤⬤◯◯Serious	Several RCTs had high or unclear risk in randomization, allocation concealment, or assessor blinding.
Inconsistency	⬤⬤◯◯Serious	Very high heterogeneity (I^2^ = 98%) across studies, even after subgroup analysis.
Indirectness	⬤⬤⬤⬤None	Direct evidence from human studies assessing histological new bone formation following ARP vs. spontaneous healing.
Imprecision	⬤⬤⬤◯Moderate	Confidence intervals crossed the null and included both clinically relevant benefit and no effect.
Publication bias	⬤⬤⬤⬤None	Egger’s test (*p* = 0.576) and funnel plot inspection did not suggest reporting bias.

**GRADE DOMAIN**

**JUDGMENT**

**EXPLANATION**

## Data Availability

All data generated and analyzed during this study—including extraction tables, risk of bias assessments, and GRADE evidence profiles—are available from the corresponding author upon reasonable request. No custom code was developed for this review. Statistical analyses were conducted using publicly available software: RevMan Web (Cochrane Collaboration) and JASP version 0.18.2.

## Supplementary Materials

The following supporting information can be downloaded at: https://www.mdpi.com/article/10.3390/dj13120556/s1, Table S1: PRISMA 2020 Checklist.

## Appendix A. Detailed Search Strategies

**Table A1:** Search Strategy for the Systematic Review.

Database	Date Searched	Search Terms	Filters Applied
PubMed/MEDLINE	15 April 2025	(“Tooth Extraction” [Mesh] OR “tooth extraction”) AND (“Alveolar Ridge Preservation” [Mesh] OR “socket preservation” OR “ridge preservation”) AND (“Bone Substitutes” [Mesh] OR “bone graft” OR “xenograft” OR “allograft” OR “synthetic bone substitute” OR “alloplast” OR “DBBM” OR “PRF” OR “CGF”) AND (“Histological Techniques” [Mesh] OR “histological analysis” OR “histomorphometric” OR “new bone formation”) AND (“Randomized Controlled Trial” [Publication Type] OR “randomized controlled trial” OR “RCT”)	Humans; English; Article type: RCT
EMBASE (Elsevier)	15 April 2025	(‘tooth extraction’/exp OR ‘tooth extraction’:ti,ab) AND (‘socket preservation’/exp OR ‘ridge preservation’:ti,ab OR ‘extraction socket’:ti,ab) AND (‘bone graft’/exp OR ‘xenograft’:ti,ab OR ‘allograft’:ti,ab OR ‘synthetic bone substitute’:ti,ab OR ‘alloplast’:ti,ab OR ‘dbbm’:ti,ab OR ‘prf’:ti,ab OR ‘cgf’:ti,ab) AND (‘histology’/exp OR ‘histological analysis’:ti,ab OR ‘histomorphometric’:ti,ab OR ‘new bone formation’:ti,ab) AND (‘randomized controlled trial’/exp OR ‘rct’:ti,ab)	Humans; English; RCT
Scopus	15 April 2025	TITLE-ABS-KEY (“tooth extraction” OR “socket preservation” OR “alveolar ridge preservation” OR “extraction socket”) AND TITLE-ABS-KEY(“bone graft” OR “xenograft” OR “allograft” OR “synthetic bone substitute” OR “alloplast” OR “DBBM” OR “PRF” OR “CGF”) AND TITLE-ABS-KEY(“histological analysis” OR “histomorphometric” OR “new bone formation”) AND TITLE-ABS-KEY(“randomized controlled trial” OR “RCT”)	English; Article; Humans
Web of Science	15 April 2025	TS = (“tooth extraction” OR “socket preservation” OR “ridge preservation” OR “extraction socket”) AND TS = (“bone graft” OR “xenograft” OR “allograft” OR “synthetic bone substitute” OR “alloplast” OR “DBBM” OR “PRF” OR “CGF”) AND TS = (“histological analysis” OR “histomorphometric” OR “new bone formation”) AND TS = (“randomized controlled trial” OR “RCT”)	Language: English; Document type: Article
Cochrane CENTRAL	15 April 2025	(“tooth extraction” OR “socket preservation”) AND (“bone graft” OR “xenograft” OR “allograft” OR “synthetic bone” OR “DBBM” OR “PRF” OR “CGF”) AND (“histological” OR “histomorphometric” OR “new bone formation”) AND (“randomized controlled trial” OR “RCT”)	Trials only
Manual Search	15 April 2025	Reference lists of included articles and relevant systematic reviews were screened	Not applicable
