# Supplementary material for: Histological Outcomes of Alveolar Ridge Preservation Versus Spontaneous Healing Following Tooth Extraction: A Systematic Review and Meta-Analysis
Source: Dent J (Basel). 2025 Nov 25;13(12):556. doi: 10.3390/dj13120556 (PMC12731776; doi:10.3390/dj13120556)
Supplement: Supplementary file 1 [file dentistry-13-00556-s001.zip › dentistry-3931637-supplementary.pdf]

Table S1. PRISMA 2020 Checklist

Section/Topic	Item #	Checklist Item	Reported (Yes/No)	Location in Document
**TITLE**	1	Identify the report as a systematic review and/or meta-analysis.	Yes	Title Page
**ABSTRACT**	2	Structured summary as per PRISMA for Abstracts checklist.	Yes	Abstract
**INTRODUCTION**				
Rationale	3	Describe the rationale for the review in the context of what is already known.	Yes	Introduction, paragraphs 1–4
Objectives	4	Provide explicit statement of the objectives or questions being addressed.	Yes	Final paragraph of Introduction
**METHODS**				
Eligibility criteria	5	Specify the inclusion and exclusion criteria.	Yes	Section 2.2
Information sources	6	List all databases, registers, websites, organizations searched.	Yes	Section 2.3
Search strategy	7	Present full search strategies for all databases.	Yes	Appendix
Selection process	8	Describe the methods used to decide whether studies met inclusion criteria.	Yes	Section 2.5
Data collection process	9	Describe how data were extracted from reports.	Yes	Section 2.6
Data items	10	List and define all outcomes and variables for which data were sought.	Yes	Section 2.7
Study risk of bias assessment	11	Describe methods used to assess risk of bias.	Yes	Section 2.8
Effect measures	12	Specify the effect measure(s) (e.g., mean difference, risk ratio) for each outcome.	Yes	Section 2.9
Synthesis methods	13	Describe methods for data synthesis, including models used and handling of heterogeneity.	Yes	Section 2.10
Reporting bias assessment	14	Describe methods used to assess risk of bias due to missing results.	Yes	Section 2.12
Certainty assessment	15	Describe methods used to assess certainty in the body of evidence (e.g., GRADE).	Yes	Section 2.13
**RESULTS**				
Study selection	16	Describe search and selection process; include flow diagram.	Yes	Section 3.1 + Figure 1 (PRISMA Flowchart)
Study characteristics	17	Cite included studies and present their characteristics.	Yes	Section 3.2 + Table 1
Risk of bias in studies	18	Present risk of bias assessments for each included study.	Yes	Section 3.3 + Table 2
Results of individual studies	19	Present summary statistics and effect estimates.	Yes	Tables 3–4; Sections 3.3–3.5
Results of syntheses	20	Present results of all statistical syntheses, heterogeneity measures, and subgroup analyses.	Yes	Sections 3.3–3.5 + Figures 2–5
Reporting biases	21	Present assessments of risk of bias due to missing results.	Yes	Section 3.4.4
Certainty of evidence	22	Present GRADE assessments.	Yes	Section 3.6 + Table 5
**DISCUSSION**				

Discussion of results	23	Interpret results in context of other evidence.	Yes	Sections 4.1–4.3
Limitations of evidence	24	Discuss limitations of included evidence.	Yes	Section 4.5
Limitations of review processes	25	Discuss limitations of review methods.	Yes	Section 4.5
Implications	26	Discuss implications for practice, policy, and future research.	Yes	Sections 4.6–4.7
**OTHER INFORMATION**				
Registration and protocol	27	Provide registration information (e.g., PROSPERO) and where the protocol is available.	Yes	Section 2.1
Support	28	Describe sources of support and role of funders.	Yes	Funding Statement
Competing interests	29	Declare any conflicts of interest.	Yes	Conflict of Interest section
Availability of data, code, and materials	30	Report data/code availability and where materials are stored.	Yes	Data Availability Statement
